# Nanoparticles and Colloids as Contributing Factors in Neurodegenerative Disease

**DOI:** 10.3390/ijerph8062200

**Published:** 2011-06-14

**Authors:** Stephen C. Bondy

**Affiliations:** Division of Occupational & Environmental Health, Department of Medicine, University of California, Irvine, Irvine, CA 92697, USA; E-Mail: scbondy@uci.edu; Tel.: +1-949-824-8077; Fax: +1-949-824-2070

**Keywords:** nanoparticles, colloids, neuroinflammation, neurodegenerative disease, immune system

## Abstract

This review explores the processes underlying the deleterious effects of the presence of insoluble or colloidal depositions within the central nervous system. These materials are chemically unreactive and can have a prolonged residence in the brain. They can be composed of mineral or proteinaceous materials of intrinsic or exogenous origin. Such nanoparticulates and colloids are associated with a range of slow-progressing neurodegenerative states. The potential common basis of toxicity of these materials is discussed. A shared feature of these disorders involves the appearance of deleterious inflammatory changes in the CNS. This may be due to extended and ineffective immune responses. Another aspect is the presence of excess levels of reactive oxygen species within the brain. In addition with their induction by inflammatory events, these may be further heightened by the presence of redox active transition metals to the large surface area afforded by nanoparticles and amphipathic micelles.

## 1. Inflammation is Elevated during Brain Aging

Excess levels of inflammation characterize the aged brain [1]. Even in the absence of clearly identifiable stimuli, the immune system of the senescent brain is chronically activated. Microglial cells are increasingly activated with aging [2,3]. The cause of this unfruitful activity is uncertain, but may be related to the finding that, after an immune reaction has been provoked in the brain, the response is more pronounced, and takes much longer to dissipate and return to basal levels than is the case with other tissues [4,5]. Thus the aged brain may be a historical repository of immune memory reflecting activating events that abated long ago.

While there is evidence of heightened inflammation in the aged brain, these changes are further augmented in many age-related or neurological diseases. These include Alzheimer’s disease [6], Parkinson’s disease [7], Huntington’s disease [8]. Other neurological disorders less clearly associated with senescence, that characteristically express neuroinflammatory changes include; the human variant of Bovine Spongy Encephalopathy and other prion diseases [9,10], and Down’s syndrome [11]. All of these involve the deposition of long-lived insoluble protein aggregates within the brain. Such intracellular proteinaceous inclusions may be constituted of misfolded proteins that are not readily guided into degradation by ubiquitin-driven proteolytic processes [12] and this can lead to their identification as non-self and thus cause prolonged activation of the innate immune system [13]. Failure of the heat shock proteins to act as effective chaperones and ensure proper folding, transport and clearance of such proteins, can lead to protein accretion and thence to microglial activation [14,15]. In a parallel manner, impaired phagocytic clearance of extracellular aggregates by microglia, can also lead to enduring inflammation [16]. Microglia are resident innate immune cells in the CNS and thus key components of the immune response. They are activated by several means, notably the Toll-like receptor (TLR) system and MAP kinases. TLR signaling also mediates the beneficial effects of microglial activation but the extended release of inflammatory signals can provoke damage associated with the development of neurodegenerative diseases. The functional outcome of TLR-induced activation of microglia in the CNS then depends on a subtle balance between protective and harmful effects [17]. Cytokines such as interleukins IL-1, IL-6 & IL-8 are primarily synthesized by activated microglia in the brain in response to pathogens and trauma. Prolonged production of these peptides can result in cytotoxicity because they recruit and activate macrophages that can produce high concentrations of reactive oxygen species. Several species of inflammatory cytokine are elevated in the brain in Alzheimer’s disease [18]. In an animal model of chronic inflammation, induced by infusion of lipopolysaccharide, there was astrogliosis as well as an increase in the levels of amyloid-β protein precursor (AβPP), IL-1, and TNF∝ mRNA levels. This was subsequently followed by hippocampal cell loss and impairment of spatial memory, all of which mirror changes seen in the Alzheimer’s disease brain [19]. This suggests that inflammation can cause some of the signs of Alzheimer’s disease rather than being merely an epiphenomenon.

The animals generally used as models in which to study brain aging are generally rodents or monkeys, neither of which normally present with Alzheimer’s or Parkinson’s diseases. Attempts have been made to overcome this by insertion of human genes associated with these diseases. Such transgenic disease models can partially express neurological deficits and neuropathology relating to these diseases.

The natural drift toward a high basal levels of immune activation found with progressive maturation can thus be further driven in a wide range of brain disorders. When mice subjected to a systemic inflammatory stimulus such as lipopolysaccharide (LPS) or tumor necrosis factor alpha (TNFα), levels of inflammatory cytokines are transiently elevated in serum and liver and these return to basal levels within 1 week. On the other hand, after LPS treatment such cytokines are elevated in central nervous tissue for over 10 months, a considerable fraction of the mouse lifespan. This is associated with both microglial activation and continuing neuronal death [20]. These findings give clues as to why the aged brain shows evidence of continuing inflammation. Earlier inflammatory events such as infections involving the whole body may be effectively remembered in the CNS. Thus inflammation can be a self-propelling mechanism of progressive neurodegeneration. This would account for the fact that many age-related neurological diseases are associated with even high levels of inflammation than those associated with normal brain aging.

Many neurological diseases are specifically associated with the aging process and are not found in younger animals. Since many of these are idiopathic and not of clear genetic origin it is likely that a host of environmental factors, by interacting with brain senescence, can initiate or promote the progression of distinct neurodegenerative disorders. An extended period of pronounced brain inflammation is directly able to damage brain structures and impair behavioral function.

## 2. The Aggregation of Intrinsic Protein Species within Nervous Tissue

There is considerable evidence that the presence of particulate materials within and around neurons can activate glial surveillance mechanisms. If these materials are irresolvable, this can lead to continued exacerbation of inflammatory responses. Many neurodegenerative diseases are associated with the extended presence of proteinaceous materials that cannot readily be removed by proteolytic enzymes. The indigestiblity of some of these may be due to their adopting the resistant β-pleating configuration. This is true of both β-amyloid within plaques and the hyperphosphorylated tau protein forming neurofibrillary tangles found in Alzheimer’s disease [21,22], aberrant prion protein found in bovine spongy encephalomyelitis [23], and β-synuclein inclusions found in Parkinson’s disease [24] as well as the mutant huntingtin protein found in Huntington’s Disease [25]. Table 1 is an incomplete listing of the aggregates and their constituent proteins, associated with various neurodegenerative conditions. Such beta-pleated proteins can be considered misfolded, and although they can lead to microglial activation [26,27], their structure does not allow them to be dispersed by heat shock proteins and the ubitiquitin-proteosome system. The constituent proteins of persistent protein aggregates found in neurological disease, are often highly ubiquitinylated suggesting ineffective attempts at degradation. However there is evidence of limited clearance of Alzheimer-associated amyloid plaques by autophagic means [28]. There is also the suggestion that under some circumstances, aggregate formation represents an effective defensive strategy [29].

Amyloid-β (Aβ) is cleaved from APP and can act in a cytokine-like manner and promote inflammatory events. For example, Aβ activates microglia causing them to act as phagocytes [30]. Such reactive glial cells not only increase nitric oxide production [31], but they also activate complement proteins, which are a key component of the inflammatory cascade [32]. Analogous to the function of interleukins, the secretion of Aβ may be an integral part of an attempted innate immune response of the CNS against pathological processes. If the provocative stimulus remains unresolved, the extended production of materials which may have beneficial value in the short term can have deleterious consequences and lead to neurodegeneration.

Prion protein (PrP) is a transmembrane glycoprotein that is constitutively expressed in both glial and neuronal cells. The misfolding and consequent aggregation of the aberrant variant of the protein (PrPsc) is thought to be responsible for spongiform encephalopathies (prion diseases) such as bovine spongy encephalopathy, Gerstmann-Sträussler-Scheinker’s syndrome and Creutzfeldt-Jakob disease. These are characterized by the extracellular deposition of the pathological form of the PrP protein accompanied by neuroinflammation, amyloid plaques, gliosis, vacuole formation and neuronal cell death [33,34]. Although its function is unknown, the normal prion protein (PrPc) incorporates copper and has superoxide dismutase activity [35]. PrPc binds copper at its N-terminal region and PrPc-deficient mice have evidence of increased levels of oxidative stress [36]. Thus, the normal form of this protein may play a role in protecting cells from oxidative damage and some of the pathology associated with PrPsc may be related to its inability to sequester copper adequately.

Inclusions within the brain, whether intracellular or extracellular, are frequently associated with evidence of generation of excess amounts of oxidant free radicals and immune activation. There is good temporal evidence to suggest a causal relation between failure to remove such inclusions and the appearance of these undesirable events. While each indigestible proteinaceous aggregate has a distinctive origin and composition, they all seem to trigger a similar extended immune response.

## 3. Non-Protein Xenobiotic Colloids and Nanoparticles

Other classes of particle of exogenous origin can deposit in the brain and are poorly cleared. These can promote cerebral inflammation and pro-oxidant changes in a manner analogous to the responses generated by aggregates of endogenous origin described above. These xenobiotic compounds (Table 2) include colloids and nanoparticles composed of inorganic materials. Inhaled mineral and organic particulates have been shown to be able to access the brain and this may occur substantially by way of the olfactory epithelium [37–39]. Many studies on the effects of air pollution on CNS disease report effects of inhaled particles in conjunction with gases, a combination typical of combustion products such as diesel fumes [40]. These complex mixtures while societally relevant will not be discussed here. However, even when particles are isolated from gaseous constituents of smoke, they can provoke an inflammatory response in brain tissues [41,42]. Several types of metal particles are known to initiate or potentiate neuroinflammation. Nanosized titanium particles can exaggerate the normal brain response to an inflammogen such as lipopolysaccharide and effect microglial activation [43]. Silver, iron oxide and manganese oxide nanoparticles may act similarly [38,44]. Even elemental carbon particles can be neuroinflammatory [45]. In addition to causing damage to neurons, an inflammatory focus induced by metallic nanoparticles near cerebral blood microvessels can increase the permeability of capillary endothelium and disrupt normal function of the blood brain barrier [46]. Although less than 0.1% of inhaled or systemically injected nanoparticles are found within the brain, they are able to enter glia and neurons and inhibit neuronal firing [47].

The exact crystalline structure of such particles and their size may be critical in determining the extent of their neurotoxicity [41,48]. Aluminum salts, which are present in colloidal form at neutral pH, are also able to elevate pro-inflammatory markers both in isolated systems and in brains of intact animals [49–51]. Aluminum suspensions in drinking water at levels resembling those found in some residential water supplies, can elevate indices of inflammation and oxidative stress in the brain [52,53].

A common feature of all such foreign materials may be their ability to attract glia to their location and, since they cannot be digested by proteolytic processes, the resulting attempted phagocytic activity is essentially ineffective. The deleterious effects of a prolonged and ineffective immune response is well illustrated in the case of lung silicosis where the persistent presence of mineral particles leads to a futile phagocytic attack by alveolar macrophages on such irresolvable foci, which ultimately leads to severe pathological changes involving inflammatory cytokines [54]. Silicosis can progress long after the cessation of exposure to the original xenobiotic stimulus. This may be comparable to the situation in the CNS when the attempted clearance of particulate matter fails. Such failure can lead to cell death and further recruitment of cells involved in the generation of inflammatory responses.

## 4. The Role of Transition Metals in Nanoparticle and Aggregate Neurotoxicity

The mechanisms by which relatively inert nanoparticles and colloids form a focus of irritation may involve their large surface area and the materials, which can be attracted to such surfaces. Copper, iron and manganese are essential metals, toxic to cells in the free form and thus generally well sequestered in tissues by their binding to specific proteins such as ferritin and ceruloplasmin. These metals are able to undergo valence changes under normal physiological conditions and this redox flux is associated with generation of damaging short-lived pro-oxidant species.

Levels of copper, zinc and iron are increased on the surface of Alzheimer’s disease senile plaques and the presence of these redox reactive metals in both plaques and neurofibrillary tangles induces hydrogen peroxide-dependent oxidation [55]. In isolated systems, aluminum potentiates the oxidative stress produced by iron [56,57]. In the presence of aluminum, iron is capable of enhanced ROS formation in protein-free liposomes with a negative charge on their outer surface. Thus, the electrostatic attraction of cations to the surface negative charge may play a role in metal-induced potentiation of ROS [58]. Both aluminum and β-amyloid peptides are not intrinsically pro-oxidant but share a similarity in their ability to stabilize iron in the ferrous form and thus enhance the Fenton reaction [59]. The ability of Fe and Cu to promote oxidative events based on their redox valence fluctuation seems to be intensified by their incomplete sequestration. Particulates and colloidal compounds may partially complex pro-oxidant metals, and thus allow them to participate in Fenton reactions for a prolonged period [60,61]. The free radical generating potency of the Fenton reaction is increased by two orders of magnitude when the iron is complexed on nanoparticle surfaces [62].

The importance of particulate-metal interactions is evident in the case of asbestos. Amosite asbestos that appears to be free of iron, can promote ROS production but this is prevented in the presence of an iron chelator [63]. This illustrates that very low amounts of metals with redox potential, bound to particulate surfaces, can initiate ROS generation. Aggregated β-amyloid peptides and high molecular weight aluminum matrices are likely to have a extensive solid/liquid interface surface area that could present an appropriate site for transition metal absorption and activation leading to an increased rate of generation of reactive oxygen species. Aβ-generated hydroxyl radical formation may be mediated by the reduction of Fe and Cu [64] and aluminum could act in a parallel manner [59]. Aluminum may also catalyze the formation of, and co-deposit together with, amyloid peptides, forming redox-active metal-complexed β-sheets [65].

It has been suggested that many age-related neurological diseases involve distortion of metal homeostasis. For example the binding of synuclein to transition metals alters its structure and aggregation propensity [66]. Excess copper and iron ingestion may accelerate age-related loss of cognition [67]. As a result, metal chelation has been suggested as a potential therapy for neurodegenerative disease [68,69].

## 5. Summary

There is a remarkable convergence of pathways activated in several neurodegenerative diseases and those activated within the brain by colloidal or particulate material of exogenous origin. While the sites of damage and types of aggregate involved can vary widely, the sequence of events triggered by their chronic presence with the brain has an extraordinary commonality. The existence of a broad range of severe neurological disorders of genetic origin, involving the accumulation of complex carbohydrates and lipids, such as galactosidoses and sphingolipidoses, further points to the inability of the brain to tolerate the prolonged accrual of insoluble materials.

The growing exposure of the population to nanoparticles from a variety of sources is a cause for concern. The major route of contact with such materials is not by way of inhalation of ambient air and fumes, but increasingly by the use of nanoparticles in consumer products, food additives and medical drugs. The cumulative effects of these exposures are unknown.

The ability of colloids and nanoparticles to initiate inflammatory events within the brain may be based on interactions between two factors:

1. Although particulate inclusions may be chemically inert, if they are a critical size, they can lead to the initiation of immune responses from glial cells. Microglia are immunocompetent and can be provoked into assuming phagocytic characteristics reminiscent of those of neutrophils and macrophages of non-nervous tissues [30]. As in the periphery, when such responses fail to clear the initiating material, this can lead to recruitment of more immune active cells and consequent increase of inflammatory activity. This cooperative interaction is a very effective antibacterial strategy but such uncontrolled innate immune responses and excessive production of inflammatory cytokines within the central nervous system (CNS) can be deleterious [70]. Infiltration of peripheral macrophages into the brain may further exacerbate such events [71].
2. The large surface area of very small particles can serve as an attractant of metal ions. When these include transition metals capable of expressing more than one valence state under physiological conditions (Fe, Cu, Mn), this forms an effective platform for Fenton-like cycling activity leading to production of reactive oxygen species [72]. It is significant that the ability of asbestos to induce oxidative damage to DNA can be suppressed by desferal, a potent iron chelator [73]. Thus the iron-sequestering capacity of this particulate plays an important role in determining its overall toxicity.

These factors could explain why a large variety of unrelated materials that are not truly soluble and are chemically rather inert, seem to be implicated in slow-progressing neurodegenerative disorders. There may thus be a link between diseases involving the progressive accretion of endogenous macromolecules in an insoluble form, and nature of the CNS damage effected by xenobiotic particulates of exogenous origin.

## Figures and Tables

**Table 1.** Proteinaceous inclusions associated with specific neurological diseases.

**Neurodegenerative disorder**	**Accumulating particles**	**Protein associated with insoluble material**

Alzheimer’s disease	Amyloid plaques, neurofibrillary tangles	β-amyloid peptides, Hyperphosphoylated tau
Dementia with Lewy Bodies (DLB)	Lewy bodies	
Parkinson’s disease	Lewy bodies	α-synuclein
Huntington’s disease	Intranuclear inclusions	huntingtin protein with expanded glutamine repeats
Spinocerebellar ataxia type 3	Intranuclear inclusion	ataxin-3 with expanded glutamine repeats
Down’s syndrome	Lewy bodies, amyloid plaques	α-synuclein, β-amyloid
Human variant of Bovine spongy encephalopathy, Creutzfeldt-Jakob disease	Amyloid plaques	Prion protein variant
Pick’s disease	Pick bodies	Hyperphosphorylated tau

**Table 2.** Particles and colloids of exogenous origin causing an inflammatory response within the CNS.

**Particle origin**	**Particle constituents**	**Reference**
Combustion products in polluted air	Organic chemicals, minerals	40,42
Combustion products in polluted air	Elemental carbon	45
Disinfection, water treatment	Silver oxide	46
Welding and mining operations	Manganese oxide	38
Medical diagnosis, therapy	Titanium oxide	43,44
Drinking water	Colloidal aluminum salts	51,52

## References

[1] Sharman EH, Bondy SC, Sharman KZ, Lahiri D, Cotman CW, Perreau VM (2007). Effects of melatonin and age on gene expression in mouse CNS using microarray analysis. Neurochem. Int.

[2] Sloane JA, Hollander W, Moss MB, Rosene DL, Abraham CR (1999). Increased microglial activation and protein nitration in white matter of the aging monkey. Neurobiol. Aging.

[3] Streit WJ, Miller KR, Lopes KO, Njie E (2008). Microglial degeneration in the aging brain-bad news for neurons?. Front. Biosci.

[4] Streit WJ (2006). Microglial senescence: Does the brain's immune system have an expiration date?. Trends Neurosci.

[5] Frank MG, Barrientos RM, Watkins LR, Maier SF (2010). Aging sensitizes rapidly isolated hippocampal microglia to LPS *ex vivo*. J. Neuroimmunol.

[6] Gorelick PB (2010). Role of inflammation in cognitive impairment: Results of observational epidemiological studies and clinical trials. Ann. N. Y. Acad. Sci.

[7] Qian L, Flood PM, Hong JS (2010). Neuroinflammation is a key player in Parkinson’s disease and a prime target for therapy. J. Neural Transm.

[8] Hsiao HY, Chern Y (2010). Targeting glial cells to elucidate the pathogenesis of Huntington’s disease. Mol. Neurobiol.

[9] Baker CA, Manuelidis L (2003). Unique inflammatory RNA profiles of microglia in Creutzfeldt-Jakob disease. Proc. Nat. Acad. Sci. USA.

[10] Riemer C, Gültner S, Heise I, Holtkamp N, Baier M (2009). Neuroinflammation in prion diseases: Concepts and targets for therapeutic intervention. CNS Neurol. Disord. Drug Target.

[11] Shapiro LA, Bialowas-McGoey LA, Whitaker-Azmitia PM (2010). Effects of S100B on Serotonergic Plasticity and Neuroinflammation in the Hippocampus in Down Syndrome and Alzheimer’S Disease: Studies in an S100B Overexpressing Mouse Model. Cardiovasc Psychiat Neurol.

[12] Salminen A, Kauppinen A, Suuronen T, Kaarniranta K, Ojala J (2009). ER stress in Alzheimer’s disease: A novel neuronal trigger for inflammation and Alzheimer’s pathology. J. Neuroinflammation.

[13] Golde TE, Miller VM (2009). Proteinopathy-induced neuronal senescence: A hypothesis for brain failure in Alzheimer's and other neurodegenerative diseases. Alzheimers Res. Ther.

[14] Wilhelmus MM, de Waal RMW, Verbeek MM (2007). Heat shock proteins and amateur chaperones in amyloid-beta accumulation and clearance in Alzheimer’s disease. Mol. Neurobiol.

[15] van Noort JM (2008). Stress proteins in CNS inflammation. J. Pathol.

[16] Linnartz B, Wang Y, Neumann H (2010). Microglial immunoreceptor tyrosine-based activation and inhibition motif signaling in neuroinflammation. Int J Alzheimers Dis.

[17] Lehnardt S (2010). Innate immunity and neuroinflammation in the CNS: The role of microglia in Toll-like receptor-mediated neuronal injury. Glia.

[18] Mandrekar-Colucci S, Landreth GE (2010). Microglia and inflammation in Alzheimer’s disease. CNS Neurol. Disord. Drug Target.

[19] Hauss-Wegrzyniak B, Lynch MA, Vraniak PD, Wenk GL (2002). Chronic brain inflammation results in cell loss in the entorhinal cortex and impaired LTP in perforant path-granule cell synapses. Exp. Neurol.

[20] Qin L, Wu X, Block ML, Liu Y, Breese GR, Hong JS, Knapp DJ, Crews FT (2007). Systemic LPS causes chronic neuroinflammation and progressive neurodegeneration. Glia.

[21] Vaden TD, Gowers SA, Snoek LC (2009). Observation of beta-sheet aggregation in a gas-phase tau-peptide dimer. J. Am. Chem. Soc.

[22] Ahmed M, Davis J, Aucoin D, Sato T, Ahuja S, Aimoto S, Elliott JI, van Nostrand WE, Smith SO (2010). Structural conversion of neurotoxic amyloid-beta(1-42) oligomers to fibrils. Nat. Struct. Mol. Biol.

[23] Ji HF, Zhang HY (2010). Beta-sheet constitution of prion proteins. Trends Biochem Sci.

[24] Heise H, Celej MS, Becker S, Riedel D, Pelah A, Kumar A, Jovin TM, Baldus M (2008). Solid-state NMR reveals structural differences between fibrils of wild-type and disease-related A53T mutant alpha-synuclein. J. Mol. Biol.

[25] Nekooki-Machida Y, Kurosawa M, Nukina N, Ito K, Oda T, Tanaka M (2009). Distinct conformations of *in vitro* and *in vivo* amyloids of huntingtin-exon1 show different cytotoxicity. Proc. Nat. Acad. Sci. USA.

[26] Veerhuis R, Boshuizen RS, Morbin M, Mazzoleni G, Hoozemans JJ, Langedijk JP, Tagliavini F, Langeveld JP, Eikelenboom P (2005). Activation of human microglia by fibrillar prion protein-related peptides is enhanced by amyloid-associated factors SAP and C1q. Neurobiol. Dis.

[27] Darnell G, Orgel JP, Pahl R, Meredith SC (2007). Flanking polyproline sequences inhibit beta-sheet structure in polyglutamine segments by inducing PPII-like helix structure. J. Mol. Biol.

[28] Town T, Nikolic V, Tan J (2005). The microglial “activation” continuum: From innate to adaptive responses. J. Neuroinflammation.

[29] Bodner RA, Outeiro TF, Altmann S, Maxwell MM, Cho SH, Hyman BT, McLean PJ, Young AB, Housman DE, Kazantsev AG (2006). Pharmacological promotion of inclusion formation: A therapeutic approach for Huntington’s and Parkinson’s diseases. Proc. Nat. Acad. Sci. USA.

[30] Zotova E, Holmes C, Johnston D, Neal JW, Nicoll JA, Boche D (2010). Microglial alterations in human Alzheimer’s disease following Ab42 immunisation. Neuropathol Appl Neurobiol.

[31] Sugama S, Takenouchi T, Cho BP, Joh TH, Hashimoto M, Kitani H (2009). Possible roles of microglial cells for neurotoxicity in clinical neurodegenerative diseases and experimental animal models. Inflamm. Allergy Drug Targets.

[32] Dheen ST, Kaur C, Ling EA (2007). Microglial activation and its implications in the brain diseases. Curr. Med. Chem.

[33] Eikelenboom P, Bate C, van Gool WA, Hoozemans JJ, Rozemuller JM, Veerhuis R, Williams A (2002). Neuroinflammation in Alzheimer’s disease and prion disease. Glia.

[34] Veerhuis R, Boshuizen RS, Familian A (2005). Amyloid associated proteins in Alzheimer’s and prion disease. Curr. Drug Targets CNS Neurol. Disord.

[35] Brown DR, Sassoon J (2002). Copper-dependent functions for the prion protein. Mol. Biotechnol.

[36] Wong BS, Liu T, Li R, Pan T, Petersen RB, Smith MA, Gambetti P, Perry G, Manson JC, Brown DR, Sy MS (2001). Increased levels of oxidative stress markers detected in the brains of mice devoid of prion protein. J. Neurochem.

[37] Peters A, Veronesi B, Calderón-Garcidueñas L, Gehr P, Chen LC, Geiser M, Reed W, Rothen-Rutishauser B, Schürch S, Schulz H (2006). Translocation and potential neurological effects of fine and ultrafine particles a critical update. Par. Fibre Toxicol.

[38] Antonini JM, Sriram K, Benkovic SA, Roberts JR, Stone S, Chen BT, Schwegler-Berry D, Jefferson AM, Billig BK, Felton CM (2009). Mild steel welding fume causes manganese accumulation and subtle neuroinflammatory changes but not overt neuronal damage in discrete brain regions of rats after short-term inhalation exposure. Neurotoxicology.

[39] Oberdörster G, Elder A, Rinderknecht A (2009). Nanoparticles and the brain: Cause for concern?. Trends Neurosci.

[40] van Berlo D, Albrecht C, Knaapen AM, Cassee FR, Gerlofs-Nijland ME, Kooter IM, Palomero-Gallagher N, Bidmon HJ, van Schooten FJ, Krutmann J (2010). Comparative evaluation of the effects of short-term inhalation exposure to diesel engine exhaust on rat lung and brain. Arch. Toxicol.

[41] Campbell A, Oldham M, Becaria A, Bondy SC, Meacher D, Sioutas C, Misra C, Mendez LB, Kleinman M (2005). Particulate matter in polluted air may increase biomarkers of inflammation in mouse brain. Neurotoxicology.

[42] Kleinman MT, Araujo JA, Nel A, Sioutas C, Campbell A, Cong PQ, Li H, Bondy SC (2008). Inhaled ultrafine particulate matter affects CNS inflammatory processes and may act via MAP kinase signaling pathways. Toxicol. Lett.

[43] Wang J, Liu Y, Jiao F, Lao F, Li W, Gu Y, Li Y, Ge C, Zhou G, Li B, Zhao Y, Chai Z, Chen C (2008). Time-dependent translocation and potential impairment on central nervous system by intranasally instilled TiO(2) nanoparticles. Toxicology.

[44] Shin JA, Lee EJ, Seo SM, Kim HS, Kang JL, Park EM (2010). Nanosized titanium dioxide enhanced inflammatory responses in the septic brain of mouse. Neuroscience.

[45] Win-Shwe TT, Mitsushima D, Yamamoto S, Fukushima A, Funabashi T, Kobayashi T, Fujimaki H (2008). Changes in neurotransmitter levels and proinflammatory cytokine mRNA expressions in the mice olfactory bulb following nanoparticle exposure. Toxicol. Appl. Pharmacol.

[46] Trickler WJ, Lantz SM, Murdock RC, Schrand AM, Robinson BL, Newport GD, Schlager JJ, Oldenburg SJ, Paule MG, Slikker W (2010). Silver nanoparticle induced blood-brain barrier inflammation and increased permeability in primary rat brain microvessel endothelial cells. Toxicol. Sci.

[47] Gramowski A, Flossdorf J, Bhattacharya K, Jonas L, Lantow M, Rahman Q, Schiffmann D, Weiss DG, Dopp E (2010). Nanoparticles induce changes of the electrical activity of neuronal networks on microelectrode array neurochips. Environ. Health Perspect.

[48] Wang B, Feng WY, Wang M, Shi JW, Zhang F, Ouyang H, Zhao YL, Chai ZF, Huang YY, Xie YN (2007). Transport of intranasally instilled fine Fe_2_O_3_ particles into the brain: Micro-distribution, chemical states, and histopathological observation. Biol. Trace Elem. Res.

[49] Campbell A, Yang Y, Tsai-Turton M, Bondy SC (2002). Pro-inflammatory effects of aluminum in human glioblastoma cells. Brain Res.

[50] Campbell A, Araujo JA, Li H, Sioutas C, Kleinman M (2009). Particulate matter induced enhancement of inflammatory markers in the brains of apolipoprotein E knockout mice. Nanosci. Nanotechnol.

[51] Bondy SC (2010). The neurotoxicity of environmental aluminum is still an issue. Neurotoxicology.

[52] Campbell A, Becaria A, Lahiri DK, Sharman K, Bondy SC (2004). Chronic exposure to aluminum in drinking water increases inflammatory parameters selectively in the brain. J. Neurosci. Res.

[53] Becaria A, Lahiri D, Bondy SC, Chen D, Hamadeh A, Li H, Taylor R, Campbell A (2006). Aluminum and copper in drinking water enhance inflammatory or oxidative events specifically in the brain. J. Neuroimmunol.

[54] Mohr C, Gemsa D, Graebner C, Hemenway DR, Leslie KO, Absher PM, Davis GS (1991). Systemic macrophage stimulation in rats with silicosis: Enhanced release of tumor necrosis factor-alpha from alveolar and peritoneal macrophages. Am. J. Respir. Cell. Mol. Biol.

[55] Rajendran R, Minqin R, Ynsa MD, Casadesus G, Smith MA, Perry G, Halliwell B, Watt F (2009). A novel approach to the identification and quantitative elemental analysis of amyloid deposits-insights into the pathology of Alzheimer’s disease. Biochem. Biophys. Res. Commun.

[56] Bondy SC, Kirstein S (1996). The promotion of iron-induced generation of reactive oxygen species in nerve tissue by aluminum. Mol. Chem. Neuropathol.

[57] Bondy SC, Guo-Ross SX, Truong AT (1998). Promotion of transition metal-induced production of reactive oxygen species formation by β-amyloid. Brain Res.

[58] Bondy SC, Guo-Ross SX, Pien J (1998b). Mechanisms underlying aluminum-induced potentiation of oxidant properties of transition metals. Neurotoxicology.

[59] Yang EY, Guo-Ross SX, Bondy SC (1999). The stabilization of ferrous iron by a toxic β-amyloid fragment and by an aluminum salt. Brain Res.

[60] HaMai D, Bondy SC, Becaria V, Campbell A (2001). The chemistry of transition metals in relation to their potential role in neurodegenerative processes. Curr. Top. Med. Chem.

[61] Bondy SC, HaMai D (2004). Oxidative basis of manganese neurotoxicity. Ann. N. Y. Acad. Sci.

[62] Voinov MA, Pagán JO, Morrison E, Smirnova TI, Smirnov AI (2011). Surface-mediated production of hydroxyl radicals as a mechanism of iron oxide nanoparticle biotoxicity. J. Am. Chem. Soc.

[63] Panduri V, Weitzman SA, Chandel NS, Kamp DW (2004). Mitochondrial-derived free radicals mediate asbestos-induced alveolar epithelial cell apoptosis. Am. J. Physiol. Lung Cell. Mol. Physiol.

[64] Smith DG, Cappai R, Barnham KJ (2007). The redox chemistry of the Alzheimer’s disease amyloid beta peptide. Biochim. Biophys. Acta.

[65] Exley CJ (2006). Aluminium and iron, but neither copper nor zinc, are key to the precipitation of β-sheets of Aβ42 in senile plaque cores in Alzheimer’s disease. J. Alzheim. Dis.

[66] Santner A, Uversky VN (2010). Metalloproteomics and metal toxicology of α-synuclein. Metallomics.

[67] Brewer GJ (2010). Risks of copper and iron toxicity during aging in humans. Chem. Res. Toxicol.

[68] Bolognin S, Drago D, Messori L, Zatta P (2009). Chelation therapy for neurodegenerative diseases. Med. Res. Rev.

[69] Horowitz MP, Greenamyre JT (2010). Mitochondrial iron metabolism and its role in neurodegeneration. J Alzheim Dis.

[70] Yang G, Meng Y, Li W, Yong Y, Fan Z, Ding H, Wei Y, Luo J, Ke ZJ (2011). Neuronal MCP-1 Mediates Microglia Recruitment and Neurodegeneration Induced by the Mild Impairment of Oxidative Metabolism. Brain Pathol.

[71] Eder C (2010). Ion channels in monocytes and microglia/brain macrophages: Promising therapeutic targets for neurological diseases. J. Neuroimmunol.

[72] Lee C, Sedlak DL (2008). Enhanced formation of oxidants from bimetallic nickel-iron nanoparticles in the presence of oxygen. Environ. Sci. Technol.

[73] Jiang L, Nagai H, Ohara H, Hara S, Tachibana M, Hirano S, Shinohara Y, Kohyama N, Akatsuka S, Toyokuni S (2008). Characteristics and modifying factors of asbestos-induced oxidative DNA damage. Cancer Sci.

